# Treatment of obstructive jaundice caused by hepatic artery pseudoaneurysm after liver transplantation: A case report

**DOI:** 10.1097/MD.0000000000018015

**Published:** 2019-12-20

**Authors:** Weijie Gao, Xinyu Li, Lei Huang

**Affiliations:** Department of Hepatobiliary Surgery, Peking University People's Hospital, Xicheng District, Beijing, P.R. China.

**Keywords:** complication, hepatic artery pseudoaneurysm, liver transplantation, obstructive jaundice

## Abstract

**Rationale:**

Despite vast improvements in technique, several complications still challenge surgeons and medical practitioners alike, including biliary and vascular complications, acute and chronic rejection, and disease recurrence.

**Patient concerns:**

A 59-year-old man was admitted to hospital on July, 2016. He had hepatitis B cirrhosis related recurrent hepatocellular carcinoma and underwent living donor liver transplantation in our hospital.

**Diagnosis:**

At the time of admission, the patient's spirit, diet, sleep, normal urine and stool, and weight did not change significantly. The test indicators are as follows: total bilirubin: 100.1 μmol/L, direct bilirubin: 65.0 μmol/L. Emergency CT in the hospital after admission showed that hepatic artery pseudoaneurysm formation after liver transplantation was observed.

**Interventions:**

This patient underwent minimal invasive endovascular treatment. The demographic, clinical, and laboratory data were collected and reviewed. He was treated successfully by endovascular stent grafting and thrombolytic treatment.

**Outcomes:**

The blood concentration of tacrolimus (FK506) was 6.3 ng/mL total bilirubin 19.6 μmol/L before discharge. The changing of total bilirubin and direct bilirubin were investigated (Fig. 1). The patient recovered well and was discharged 2 weeks later. The patient is doing well and regularly followed up.

**Lessons:**

Coil embolization of aneurysmal sac or placement of a stent graft is a minimally invasive alternative to surgery and definitively excludes a bleeding hepatic artery pseudoaneurysm. This technique can be considered as an effective treatment option for hepatic artery pseudoaneurysm instead of a difficult surgical repair.

## 1 Introduction

Orthotopic liver transplantation (OLT), which is a procedure that has continued to evolve since it was first attempted in 1967,^[1]^ is accepted worldwide as a definitely standard therapy for end-stage liver disease.^[2,3]^ Despite vast improvements in technique, several complications still challenge surgeons and medical practitioners alike, including biliary and vascular complications, acute and chronic rejection, and disease recurrence.^[4–7]^

Biliary complications (BCs) remain one of the most outstanding factors influencing the long-term results after OLT. This is the main factor of late complications besides the recurrence of hepatitis C, and primary sclerosing cholangitis (PSC), and the occurrence of de novo diseases, like diabetes mellitus, and kidney impairment as a result of drug side effects.^[8,9]^ BCs can be classified according to their anatomic site, the time of occurrence, and by the etiology factor leading to this complication.^[10]^ Hepatic artery pseudoaneurysm (HAP), which is one of vascular complications after liver transplantation that reportedly occur in 6% to 10% of cases and often require urgent surgical management and frequently result in graft failure, usually necessitating re-transplantation, and can lead to patient death, is reported to occur in 1% to 2% of OLT patients and could cause biliary obstruction that is associated with significant morbidity and mortality. Mortality rates for ruptured HAP have been reported to be as high as 69%.^[11,12]^

HAP may be asymptomatic and detected during imaging evaluation for other reasons. They may present with nonspecific symptoms such as hemobilia, a falling hemoglobin level, unexplained fever, or graft dysfunction or may manifest with gastrointestinal (GI) bleeding or hemoperitoneum, which is often presented as profound shock. Early diagnosis requires a high level of suspicion and close monitoring.^[13,14]^ With early diagnosis, a number of treatment options exist to prevent life threatening hemorrhage and for graft salvage. Existing treatment options that allow for preservation of the arteria flow into the graft are surgical resection and revascularization, and catheter-based minimal invasive endovascular treatments such as coil embolization and stent grafting.^[15,16]^

We have report a series of cases of hepatic artery pseudoaneurysms following OLT that were treated successfully by endovascular stent grafting and thrombolytic treatment in our institute. Between January 2014 and July 2018, a total of 68 patients underwent living donor liver transplantation at our institute. Among these patients, 3 patients (4.41%) developed hepatic artery pseudoaneurysms after the transplantation. One of them demonstrated biliary obstruction symptoms which were rarely caused by HAP. This patient underwent minimal invasive endovascular treatment. The demographic, clinical, and laboratory data were collected and reviewed.

## 2 The case

**Figure 1:**
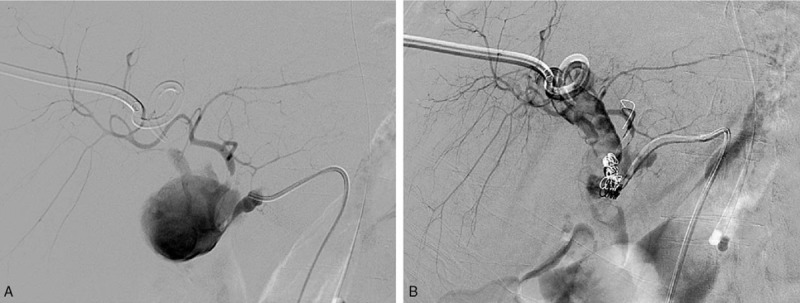
Changing of test indicators during the operation. (A) Total bilirubin and (B) direct bilirubin decreased after the operation.

**Figure 2:**
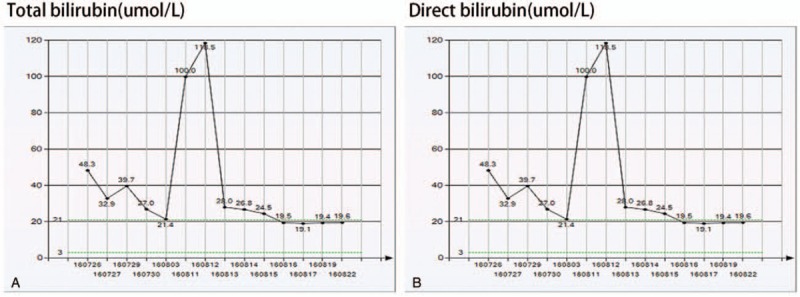
Hepatic artery angiography for the case. (A) Pseudoaneurysm at the anastomotic site and the right hepatic artery. (B) Pseudoaneurysm disappeared after placement of stent graft.

A 59-year-old man was admitted to hospital on July, 2016. He had hepatitis B cirrhosis related recurrent hepatocellular carcinoma and underwent living donor liver transplantation in our hospital. The patient was received postoperative anti-rejection, liver protection, choleretic, and improved circulation therapy. He was transferred to our department because of yellow skin and sclera and bleeding around the tube T. At the time of admission, the patient's spirit, diet, sleep, normal urine and stool, and weight did not change significantly. The test indicators are as follows: total bilirubin: 100.1 μmol/L, direct bilirubin: 65.0 μmol/L. Emergency computed tomography (CT) in the hospital after admission showed that hepatic artery pseudoaneurysm formation after liver transplantation was observed (Fig. 2A). The patients received emergency digital subtraction angiography (DSA), and an intraluminal insomnia was placed in the 5 × 25 mm and 5 × 50 mm stent grafts (viabahn) in the hepatic artery of the lesion. The 4 × 40 mm balloon was used for posterior dilatation and re-imaging (Fig. 2B). The position of the stent was good, the original pseudoaneurysm and anastomotic stenosis disappeared, and the right hepatic artery blood flow was slow, considering acute thrombosis. Intrahepatic right artery was injected with r-tPA 20 mg thrombolysis. After thrombolysis, the angiography showed that the right hepatic artery blood flow was smooth. After the operation, the angiography showed that the hepatic artery blood flow was smooth and the HAP disappeared. After that the urokinase 25w unit and recombinant Human Tissue Plasminogen Activator for Injection (rt-PA) 50 mg were infused as assisted anticoagulation. After 2 days the doppler ultrasound showed that the hepatic artery the blood flow spectrum was not detected. Emergency intervention with DSA showed that after the hepatic artery stent implantation, no contrast agent was passed through the stent. Considering stent thrombosis, the super-sliding guide wire was combined with the contrast catheter to try to enter the distal part of the hepatic artery through the stent thrombus. DSA showed that illing defect could be seen in the stent. Microcatheter is used and r-tPA 30 mg is injected into the stent through microcatheter. Repeated DSA showed that the filling defect in the original stent disappeared, the distal branch of the hepatic artery develops well. 3D angiography showed that the stent was severely stenotic in the distal hepatic artery. Considering the thrombus formation in the stent was related to the hepatic artery stenosis. The sacral catheter was placed in a 260 cm 0.035″ hardened guide wire, and the 7F long sheath was guided along the guide wire into the stent. A stent-release system was introduced through the long sheath, and a 5 × 15 mm ball-expanded stent (Boston) was placed in the stenosis of the hepatic artery. The angiography showed that the stent was in good position, the original hepatic artery stenosis disappeared, and the blood flow in the liver was smooth. There was no discomfort in the T tube. The blood concentration of tacrolimus (FK506) was 6.3 ng/mL total bilirubin 19.6 μmol/L before discharge. The changing of total bilirubin and direct bilirubin were investigated (Fig. 1). The patient recovered well and was discharged 2 weeks later. The patient is doing well and regularly followed up.

## 3 Discussion

Hepatic artery complications after liver transplantation can lead to ischemia of the liver graft, which can result in graft loss or patient death. The clinical features depend on the type of complications (thrombosis, stenosis, pseudoaneurysm), timing of occurrence (early or late presentation after OLT), and promptness of the diagnosis.^[17–19]^ PA of the hepatic artery after LT is rare, with an incidence of about 2%, but is potentially catastrophic. The most common presentation of PA of the hepatic artery is rupture with massive hemorrhage.^[20–22]^ It may rupture into the adjacent liver, portal, or biliary system, or directly into the abdominal cavity. The manifestations include intra-abdominal bleeding, gastrointestinal hemorrhage, hemobilia, sometimes accompanied by jaundice or fever, hypotension, or death.

Biliary complications are considered the Achilles heel of LT because of their frequency and their lethal potential on the eventual survival of both graft and recipient.^[23,24]^ The overall incidence of biliary complications after LT appears to be decreasing, from the 30% in the pioneering years to about 20% in the 1980s, and now to the current levels of 15%, except for an occasional series with a very low incidence.^[25,26]^ In the present study, we investigated a patients with symptoms of biliary obstruction resulting from compression of HAP. Coil embolization of the aneurismal sac or exclusion of the pseudoaneurysms by stent graft has been reported as an important and the most effective treatment option even in an emergency setting. If the neck of pseudoaneurysm is broad, endovascular stent grafting to exclude the pseudoaneurysm proves to be the most effective treatment option. This technique may be performed immediately following the diagnostic angiography, and has the unique advantage of completely excluding the pseudoaneurysm without injecting embolic agents into the aneurysm and concomitantly preserving the arterial blood flow to the graft. Until now, limited evidence suggests that stenting of the hepatic artery pseudoaneurysm during an acute hemorrhage can be successfully performed in elective as well as emergency setting. This prompted us to use endovascular stent grafting as the primary treatment for hepatic artery pseudoaneurysms. In this study, stent grafting was applied and the thrombolytic and anticoagulant drugs were used after the operation. At the same time, this technique effectively relieves biliary obstruction.

In conclusion, hepatic artery pseudoaneurysm is a rare vascular complication after liver transplantation, especially those with atypical clinical manifestations. Coil embolization of aneurysmal sac or placement of a stent graft is a minimally invasive alternative to surgery and definitively excludes a bleeding hepatic artery pseudoaneurysm. This technique can be considered as an effective treatment option for hepatic artery pseudoaneurysm instead of a difficult surgical repair.

## Author contributions

**Conceptualization:** Weijie Gao, Lei Huang.

**Data curation:** Weijie Gao, Xinyu Li.

**Formal analysis:** Xinyu Li.

**Methodology:** Weijie Gao.

**Project administration:** Weijie Gao.

**Resources:** Weijie Gao.

**Supervision:** Lei Huang.

**Validation:** Lei Huang.

**Writing – original draft:** Weijie Gao.

**Writing – review & editing:** Lei Huang.
